# Evaluation of Motor Control Through Functional Movement Patterns of the Lumbar Spine Among Elite Special Forces Operators: A Pilot Study

**DOI:** 10.3390/healthcare13162050

**Published:** 2025-08-19

**Authors:** Rita Hansdorfer Korzon, Jolanta Szamotulska, Piotr Wąż, Maciej Śliwiński, Jakub Ławnicki, Rafał Studnicki

**Affiliations:** 1 Department of Physiotherapy, Faculty of Health Sciences with the Institute of Maritime and Tropical Medicine, Medical University of Gdańsk, 7 Dębinki Street, 80-211 Gdańsk, Poland; rita.korzon@gumed.edu.pl (R.H.K.); jolanta.szamotulska@gumed.edu.pl (J.S.); sliwinskim@gumed.edu.pl (M.Ś.); jklawnicki@gumed.edu.pl (J.Ł.); 2 Department of Nuclear Medicine, Medical University of Gdańsk, Tuwima 15, 80-210 Gdańsk, Poland

**Keywords:** motor control, lumbar spine, biomechanics, soldiers

## Abstract

**Background**: A comprehensive physical therapy process includes prevention against musculoskeletal overload syndromes. Monitoring the occurrence of motor control disorders is one of the tools for preventing overload syndromes of the musculoskeletal system and consequent injuries. Assessing motor control and preventive actions can contribute to minimizing the risk of a soldier being removed from duty, reducing the likelihood of injury and thus preventing job loss. The aim of the study was to evaluate directional control of the lumbar spine using the dissociation tests included in the Kinetic Control physiotherapy method. This physiotherapeutic method is used to identify and assess the occurrence and therapy of motor control disorders, including uncontrolled movement in the locomotor system. **Methods**: Twenty-three soldiers (40.26 ± 4.5 age) from special units of the Polish Armed Forces were qualified for a one-time assessment. The research methods included the evaluation of motor control using dissociation tests based on the physiological method of kinetic control. The control of the lumbar spine in the directions of flexion, extension, and rotation during hip joint movements was evaluated. Uncontrolled movement was understood as the inability to maintain a stationary lumbar spine in a neutral position during specific directions of hip joint movement included in the tests. **Results**: The survey showed that the area of pain reported by the operators was the lumbar spine in the last three months. 69.57% of the respondents indicated that this area was the site of their complaints. The results of the motor examination showed statistically significant test results (*p*-value < 0.0001) indicating the presence of motor control disorders in the form of uncontrolled movement of the lumbar spine in control tests for flexion, rotation, and extension. **Conclusions**: The main results of the present study showed the appearance of interference with the functional movement patterns of the lumbar spine in a group of special unit operators. Impaired control of movement was observed in the direction of flexion, rotation, and lumbar extension, which may be potentially associated with the generation of lumbar spine pain syndromes.

## 1. Introduction

The primary goal of motor control (MC) is to maintain body stability during free movements [1]. Factors such as physical and psychosocial conditions or the type of work performed are potentially associated with impaired motor control, including the musculoskeletal system [2]. In cases where uncontrolled movement disorders occur, a mechanism of inhibition or alteration of motor control is induced [3]. Therefore, members of the special forces are also exposed to high levels of stress on the musculoskeletal system while on duty. Both the preparatory phase of the selection process and the various long and intensive training periods, as well as the performance of duty in combat units, significantly affect the musculoskeletal system of operators [4]. The long and varied process of intensive training, such as running with full equipment or jumping with parachutes, leads to musculoskeletal overload syndromes. We can read in the literature that this affects about 20% of the special forces operators of the US Army per year [5] and 68% of injuries occur during the training process of the soldiers [6]. In their work, Stannard J. and Fortington L. identify the lumbar spine area as one of the three most common areas of localized discomfort along with the knee and ankle joints [6]. The most commonly reported lumbar motor control impairments include delayed activation of the deep spinal and lumbar muscles [7]. Abnormal motor control is defined as the processing of erroneous information by the central nervous system to control posture and generate coordinated movements [8].

An essential role in the evaluation of motor control based on the evaluation of directional control of movement may be part of secondary prevention. However, the early application of uncontrolled movement reeducation elements can also be beneficial in the primary prevention of injury and overload syndromes [9,10]. It can also affect muscle compensation, which will translate into precision in performing a task such as shooting [11,12].

When evaluating the lumbar spine, movement dissociation is defined as the ability to perform a specific movement at the hip joint in a specific direction while maintaining the lumbar spine in a neutral position [13]. Existing studies have focused mainly on young military [14]. There is a lack of reports in the available literature related to the assessment of lumbar spine directional motor control by Kinetic Control dissociation tests in a group of special units operators. This publication is an attempt to fill the existing gap in the literature and is potentially related to improving physiotherapeutic care in this group.

The aim of the study was to evaluate directional control of the lumbar spine using the dissociation tests included in the Kinetic Control physiotherapy method.

Addressing the study of soldiers with several years of experience may be necessary to assess health and future injuries. The present study is a pilot study to look for possible motor control deficits in a group of special unit operators, the identification of which should be a prelude to considering the topic of motor control and also to implementing elements of motor control reeducation into the standards of medical care for a group of special forces operators. The introduction of training elements in the reeducation of motor control disorders in the evaluated group may contribute to the primary and secondary prevention of overload and injury in the lumbar spine. This may be associated with a reduction in the work absenteeism of special unit operators and lower medical costs in the future.

## 2. Materials and Methods

### 2.1. Ethics

The Independent Bioethics Committee for Scientific Research at the Medical University of Gdańsk approved this study on 14 June 2022 (Resolution No. KB/206/2022). The study was conducted between October 2022 and December 2022. The study protocol was thoroughly explained to all participants, and written informed consent was obtained from each volunteer, clearly stating that he could withdraw from the study at any time. The study was carried out according to ethical standards for research involving human participants, as summarized in the Declaration of Helsinki.

### 2.2. Participants

**Figure 1:**
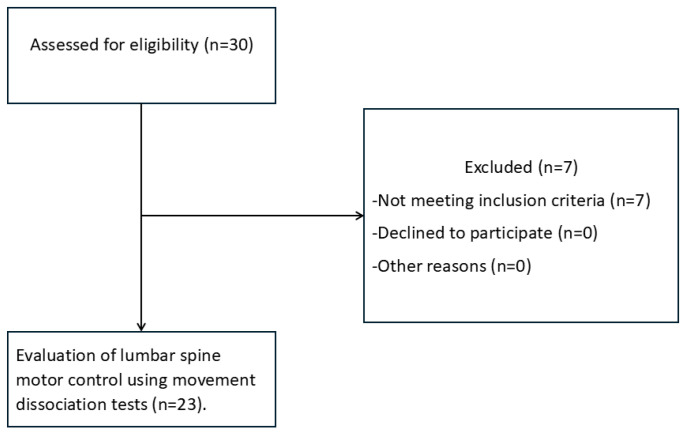
Flowchart of the participants.

The study initially included 30 soldiers, of whom 7 were excluded because they did not meet the passive range of motion criteria for the hip joints. Ultimately, 23, male soldiers from Polish Armed Forces Special Operations Units were qualified for a one-time assessment. These soldiers had previously served or are currently serving in combat units. Figure 1 presents the flow chart of the study participants.

The inclusion criteria were current or previous service in Special Forces combat units (minimum of one year in combat teams), aged between 20 and 60 years, and male sex. Additional requirements included a full range of motion of the lumbar spine and hip joint sufficient to complete the selected motor control tests, no lumbar spine injuries in the past 12 months, no damage to the nervous system and proper neurological functioning, stable mental health, and absence of comorbid conditions.

Exclusion criteria included a history of fractures, dislocations, or surgeries involving the lumbar spine or hip joints, current acute episodes of lumbar pain, injuries to the spine or hip joints within the last 12 months, and the presence of comorbid diseases. Table 1 shows the basic demographics of the study group.

### 2.3. Evaluation Protocol

#### Card of Basic Information and Location of Pain Complaints

As part of the subject examination, information was collected on basic sociodemographic data, such as age, weight, height, and duration of service in special forces combat units. Information on the operator’s medical history was also collected to assess whether the subject met the established exclusion criteria for the study. The next step was for the subject to indicate the areas of the body where he had experienced pain in the last three months. Due to the premise of the study, the results did not differentiate between pain on the right/left side or bilateral pain.

### 2.4. Assessment of Motor Control Using Dissociation Tests

#### 2.4.1. The Testing Procedure and Evaluation Method

Motor control was tested using dissociation tests included in the kinetic control physiotherapy method used to evaluate the area of the motor system. These tests are an assessment tool as part of a functional physiotherapeutic examination that allows identification of the occurrence of uncontrolled movement in the lumbar spine area during hip joint movements. Excessive uncontrolled movement of the lumbar region during hip joint movements with deficits in muscle activation present can contribute to overloading of musculoskeletal structures. Identification of directional control errors using the tests used in the evaluation makes it possible to subsequently reeducate motor control deficits in the therapeutic process and thus improve motor control in the lumbar spine area and consequently a higher level of protection for the musculoskeletal system [9,13,15,16].

The tests were carried out in the Movement Laboratory of the Department of Physiotherapy of the Medical University of Gdansk by a trained researcher who received certified training in the Kinetic Control method. An investigator evaluated the volunteers each time and controlled the correct neutral position by positioning the volunteer’s pelvis so that the line between the upper anterior iliac spine and the pubic tubercle was vertical. During the test procedure, after instruction, the subject’s task was to maintain the lumbar spine in a neutral position while moving the lower limb in a fixed direction.

To properly control the alignment of the lumbar spine in the sitting and standing positions, an inclinometer was used according to the guidelines developed by the manufacturer based on the instructions of a Saunders^®^ digital inclinometer (The Saunders Group, Inc., Chaska, MN, USA) [17]. For the control in the supine position, the evaluation was performed with a rigid patch. To control the position of the lumbar spine in the neutral position, the inclinometer reader was zeroed in the horizontal position and then applied to a palpably defined lumbosacral transition (LS point). Before performing motor control tests, the researcher evaluated active and passive mobility of the hip and knee joints as a condition of being able to perform the tests. An average range of motion was established for each volunteer, helping avoid additional compensations that could disrupt the test. This determination prevented the subject from participating in the motor control test according to the test methodology. Each of the test volunteers was evaluated in two aspects: quantitative and qualitative. The quantitative component consisted of performing the indicated active movement with the lower limb within the planned range of motion while maintaining the active neutral position of the lumbar spine. The qualitative aspect consisted of evaluating the presence of compensations described in the literature while fulfilling the quantitative component of the assessment [13].

The possibility of obtaining results during the survey is recorded below.

V, V, completion of the quantitative and qualitative aspects. This result indicates that the test subject is able to perform the set movement at the hip joint specified in the test without losing the neutral position of the lumbar spine. The movement is subjectively stated by the test person as easy to perform, and there are no compensations.

V, X, the fulfillment of the quantitative criterion with the non-fulfillment of the qualitative criterion. This result indicates that the test subject is able to perform the set movement at the hip joint specified in the test without losing the neutral position of the lumbar spine. However, the movement is subjectively stated by the subject as difficult to perform, and there are compensations in the area of the lumbar–pelvic–hip complex.

X, X, failure to meet the quantitative and qualitative criteria. This result indicates that the subject is unable to perform the set hip joint movement specified in the test without losing the neutral position of the lumbar spine.

Failure to meet both requirements is equivalent to recognizing the presence of uncontrolled movement [13].

#### 2.4.2. Selected Motor Control Assessment Tests

Below are descriptions of the lumbar directional control tests used.

Evaluation in a supinated position.

1. Flexion control test of the lumbopelvic complex.

**Figure 2:**
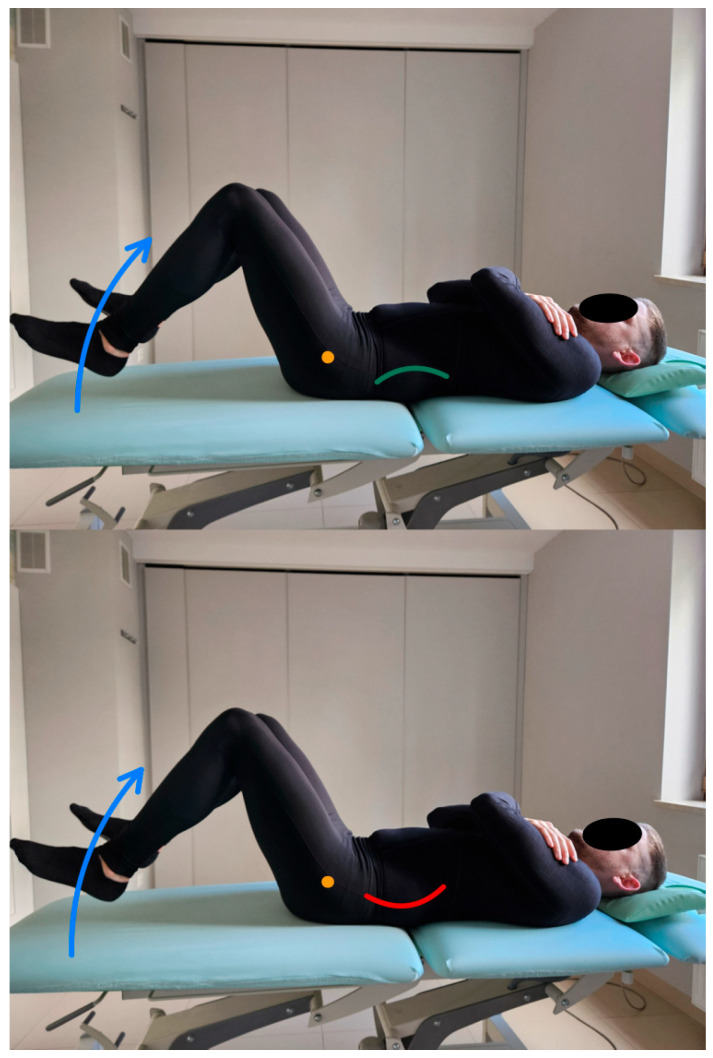
Flexion control test of the lumbopelvic complex (left side correct performance VV: hip flexion movement with maintenance of a neutral position of the lumbar spine—green line, right side incorrect performance XX: hip flexion accompanied by uncontrolled flexion of the lumbar spine—red line. The orange dot marks the greater trochanter of the femur). The blue arrow indicates the direction of lower limb movement. This direction is also indicated in the test descriptions.

In this test, the subject is supine, the lower limbs bent at the hip joints to 45 degrees, and the knee joints to 90 degrees. The pelvis and lumbar spine are placed in a neutral position in the initial position for the test. During the assessment, the test subject will perform a hip flexion movement to 90 degrees while lifting the lower extremities, keeping the pelvis and lumbar spine stationary. The therapist observes and palpates the pelvic alignment during the test [13]. On the other hand, the subject reports the appearance of impaired control of movement with the application of a rigid patch taped to the lumbosacral spine from the height (posterior superior iliac spine) of the SIPS (spina iliaca posterior superior) along the line of the spinous processes of the L segment [18,19]. Figure 2 shows the flexion control test of the lumbopelvic complex and the execution of the movement for the described test.

2. Lumbar extension control test.

**Figure 3:**
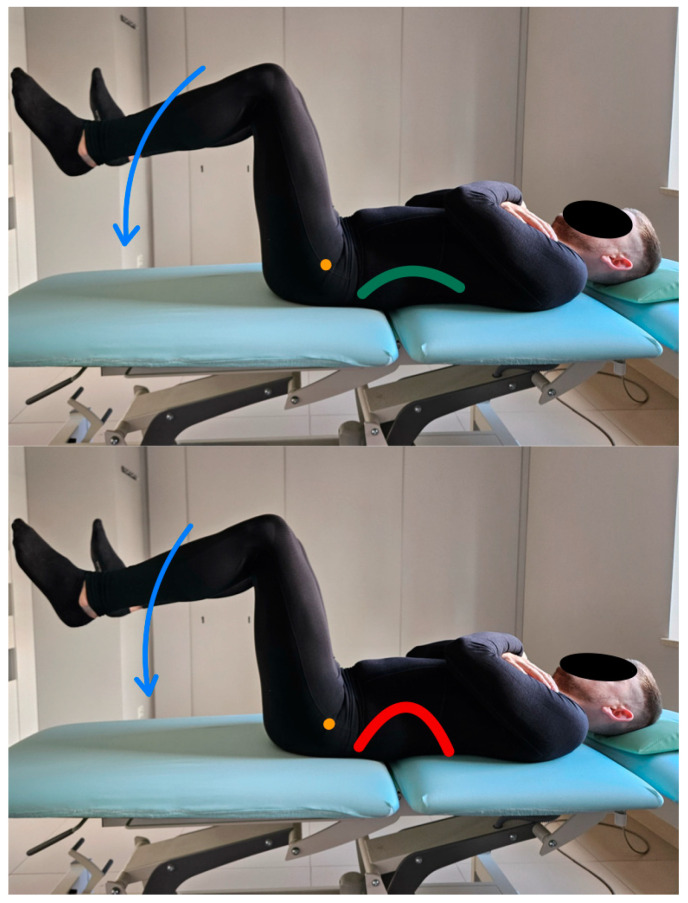
Lumbar extension control test (left side correct performance VV: hip extension movement with maintenance of a neutral position of the lumbar spine—green line, right side incorrect performance XX: hip extension with accompanying uncontrolled lumbar spine extension—red line. The orange dot marks greater trochanter of the femur).

In this test, the test subject is in a supine position, the lower limbs are raised to a flexion position at the hip joints to 90 degrees, and the knee joints to 90 degrees. The pelvis and lumbar spine are placed in a neutral position at the start of the test. During the assessment, the subject must perform a hip flexion movement at 45 degrees of flexion, lowering the lower limbs so that the feet do not rest on the ground while keeping the pelvis and lumbar spine stationary [13]. The therapist observes and, using palpation, controls pelvic alignment during the test. On the other hand, the test subject reports impaired control of movement by applying a rigid patch taped to the lumbosacral region from the height of the SIPS (spina iliaca posterior superior) along the line of the spinous processes of the L segment [18,19]. Figure 3 shows the lumbar extension control test and the execution of the movement for the described test.

3. Lumbopelvic complex rotation control test.

**Figure 4:**
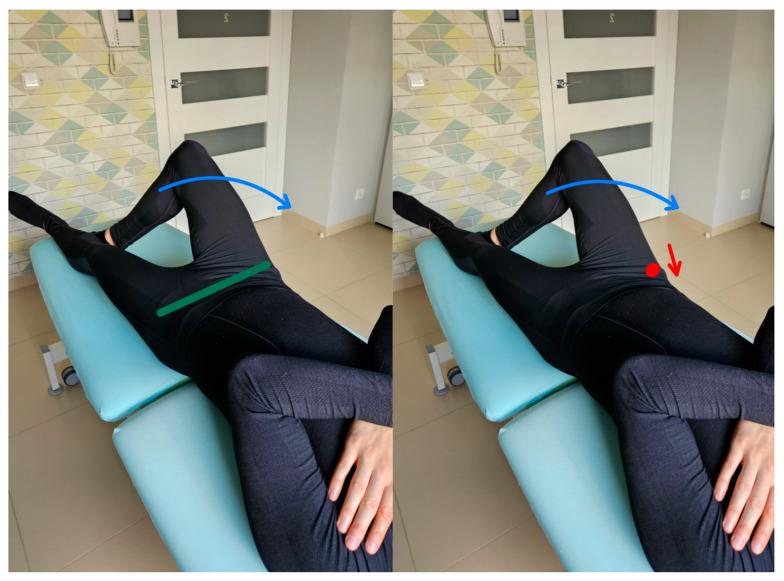
Lumbopelvic complex rotation control test (left side, correct performance VV: hip joint external rotation and abduction with keeping the anterior superior iliac spines at one height—green line, right side incorrect performance XX: lowering the right anterior superior iliac spine during the movement (red arrow) of abduction and external rotation at the right hip joint—red dot).

In this test, the test subject is in a supine position; One lower limb is straight, and the other is bent at the knee and hip joints so that the foot of this limb is at the knee level of the straight limb and in contact with it. The pelvis and lumbar spine are placed in a neutral position at the start of the test. During the assessment, the test subject is asked to perform a movement to lower the knee to the side by flexing and externally rotating the hip joint while keeping the pelvis stationary. The therapist observes and uses palpation to control pelvic alignment during the test through SIPS (spina iliaca posterior superior) [18,19]. Figure 4 shows the lumbopelvic complex rotation control test and the execution of the movement for the described test.

Assessment of the sitting position.

4. Control test of lumbar spine flexion.

**Figure 5:**
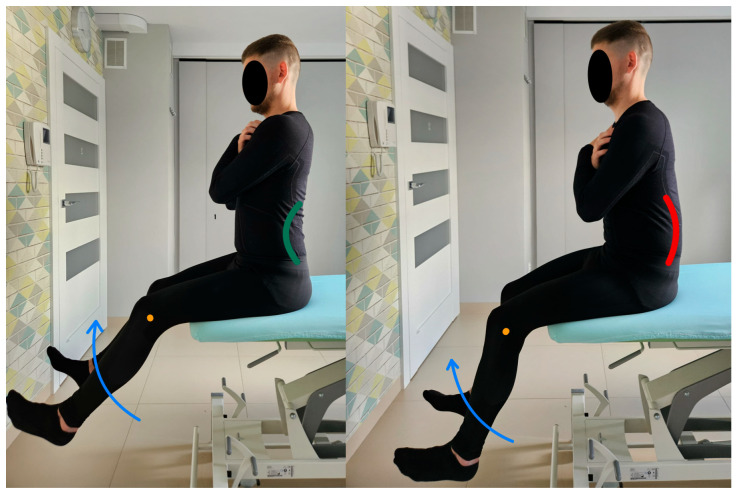
Control test of lumbar spine flexion (left side correct performance VV: straightening of both knee joints with maintenance of neutral position of the lumbar spine—green line, right side incorrect performance XX: straightening of both knee joints accompanied by uncontrolled flexion of the lumbar spine—red line. The orange dot marks the knee joint).

In this test, the test subject is in a sitting position without foot support (the angle between the torso and the thigh is 90 degrees). The pelvis and lumbar spine are placed in a neutral position in the starting position for the test. During the assessment, the test subject is expected to straighten both knee joints from 90 degrees of flexion to approximately 10–15 degrees of flexion while keeping the lumbar spine stationary and not tilting the trunk back. The therapist monitors lumbosacral alignment during the test by applying the Saunders^®^ digital inclinometer to the spinous processes of the L5–S2 vertebrae. Any change in the value read on the device was taken as the appearance of uncontrolled movement [20]. Figure 5 shows the control test of lumbar spine flexion and the performance of the movement for the described test.

5. Rotation control test of the lumbopelvic complex.

**Figure 6:**
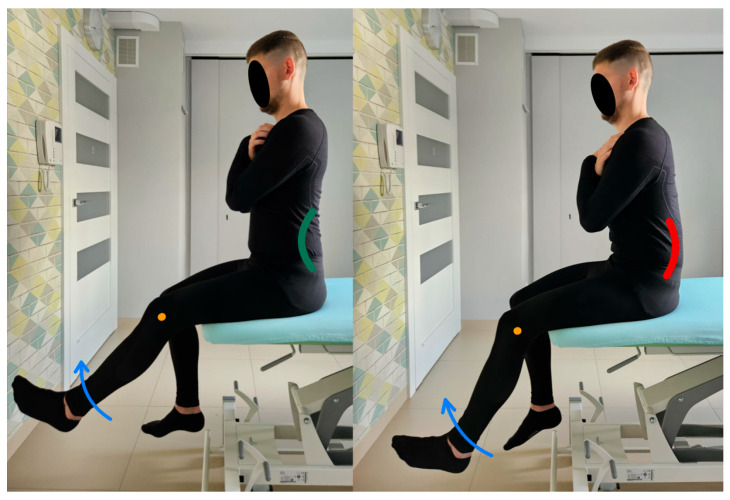
Rotation control test of the lumbopelvic complex (left side correct performance VV: straightening of the knee joint with maintenance of a neutral position of the lumbar spine—green line, right side incorrect performance XX: straightening of the knee joint accompanied by uncontrolled rotation of the lumbar spine—red line. The orange dot marks the knee joint).

In this test, the test subject is in a sitting position without foot support (the angle between the torso and the thigh is 90 degrees). The pelvis and lumbar spine are placed in a neutral position in the starting position for the test. During the assessment, the test subject is to perform a single knee joint extension from 90 degrees of flexion to approximately 10–15 degrees of flexion while keeping the lumbar spine stationary, without tilting the trunk back and without rotating the lumbar–pelvic complex [6]. Information about the appearance of impaired control of movement was obtained by applying a rigid patch taped to the lumbosacral region section from the height of the SIPS (spina iliaca posterior superior) along the line of the spinous processes of the L segment [20]. Figure 6 shows the rotation control test of the lumbopelvic complex and the performance of the movement for the described test.

6. Lumbar spine flexion control test.

**Figure 7:**
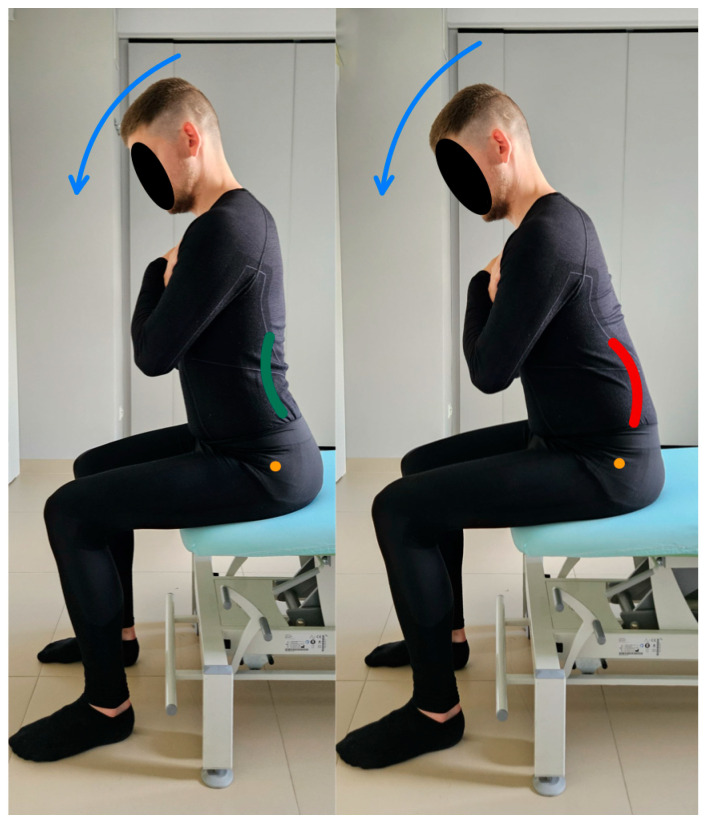
Lumbar spine flexion control test (left side correct performance VV: bending of the trunk through hip flexion movement with maintenance of a neutral position of the lumbar spine—green line, right side incorrect performance XX: bending of the trunk through hip flexion accompanied by uncontrolled flexion of the lumbar spine—red line. The orange dot marks the greater trochanter of the femur).

The subject is sitting (the angle between the torso and thigh is 90 degrees), with the feet resting on the floor. The pelvis and lumbar spine are placed in a neutral position at the beginning of the test. During the assessment, the subject tilts the torso to within 30 degrees by moving in a hip flexion movement while keeping the lumbar spine stationary [13]. The therapist monitors lumbosacral alignment during the test by applying the Saunders^®^ digital inclinometer to the spinous processes of the L5–S2 vertebrae. Any change in the digital output indicates uncontrolled movement [20]. Figure 7 shows the lumbar spine flexion control test and the performance of the movement for the described test.

Evaluation in standing position.

7. Lumbar spine flexion control test.

**Figure 8:**
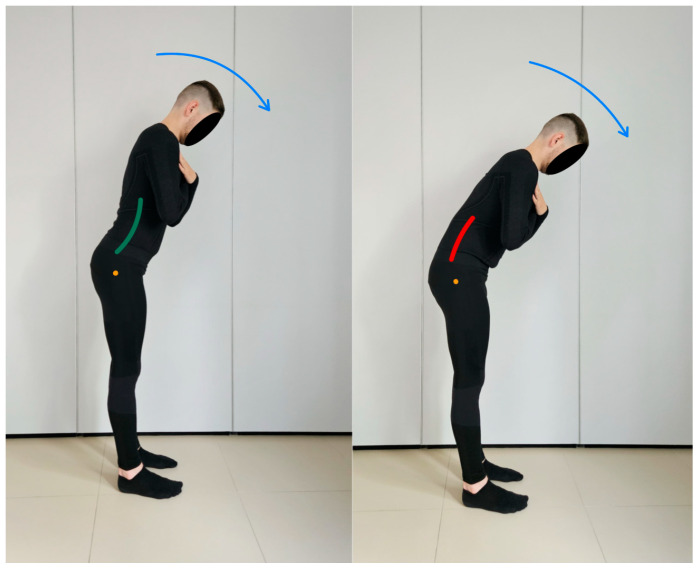
Lumbar spine flexion control test (left side correct performance VV: bending of the trunk through hip flexion movement with maintenance of a neutral position of the lumbar spine—green line, right side incorrect performance XX: bending of the trunk through hip flexion accompanied by uncontrolled flexion of the lumbar spine—red line. The orange dot marks the greater trochanter of the femur).

In this test, the subject is in a standing position. The pelvis and lumbar spine are placed in a neutral position at the beginning of the test. During the assessment, the test subject is asked to tilt the trunk within 50 degrees using a hip flexion movement while keeping the lumbar spine stationary [13]. The therapist visually monitors the movement of the lumbar–pelvic complex during the test by applying a Saunders^®^ digital inclinometer to the spinous processes of the L5–S2 vertebrae. Any change in device value indicates the appearance of uncontrolled movement [20]. Figure 8 lumbar spine flexion control test shows the performance of the movement for the described test.

### 2.5. Statistical Analysis

The Shapiro–Wilk test was used to assess whether the values of the quantitative variables were drawn from a normally distributed population. Means and standard deviations for normally distributed variables were reported. Results were expressed as the number or percentage of participants per category for qualitative variables. The results of the motor control evaluation (from the dissociative kinetic control tests) were categorized into three groups. It was assumed that 95% of the participants should fall into the VV group, 4.75% in the VX group, and 0.25% in the XX group.

The chi-square (χ^2^) test (2) was used to test for conformity to the expected distribution. When *p*-values could not be reliably determined from the asymptotic χ^2^ distribution, Monte Carlo simulations with 10,000 iterations were used. Statistical analyses were conducted using functions of the R programming environment version 4 [21]. The significance threshold was set at α = 0.05.

## 3. Results

### 3.1. Musculoskeletal Pain Complaints Among Operators

Regarding the areas of pain reported, the lumbar spine was the most frequently mentioned site of discomfort in the last three months. In fact, 69.57% of the operators reported experiencing pain in the lumbar spine during that period. The results are shown in Table 2.

### 3.2. Results of the Lumbar Spine Motor Control Testing

Lumbar spine flexion control test in supine position—hip flexion, *n* = 23. The test results showed failure to meet quantitative and qualitative criteria (X, X) in 11 participants, failure to meet the qualitative aspect while meeting the quantitative aspect (V, X) in 8 participants, and satisfaction of both assessment aspects (V, V) in 4 participants (*p*-value < 0.0001). The results are presented in Table 3.

Control test of lumbar spine extension in the supine position—hip extension, *n* = 23. The test results showed failure to meet quantitative and qualitative criteria (X, X) in 14 participants, failure to meet the qualitative aspect while meeting the quantitative aspect (V, X) in 4 participants, and satisfaction of both assessment aspects (V, V) in 5 participants (*p*-value < 0.0001). The results are presented in Table 3.

Lumbar rotation control test in the supine position—lowering the knee, right leg, *n* = 23. The test results showed failure to meet quantitative and qualitative criteria (X, X) in 7 participants, failure to meet the qualitative aspect while meeting the quantitative aspect (V, X) in 4 participants, and completion of both assessment aspects (V, V) in 12 participants (*p*-value < 0.0001). The results are presented in Table 3.

Lumbar rotation control test in the supine position—lowering the knee, left leg, *n* = 23. The test results showed failure to meet quantitative and qualitative criteria (X, X) in 9 participants, failure to meet the qualitative aspect while meeting the quantitative aspect (V, X) in 4 participants, and satisfaction of both assessment aspects (V, V) in 10 participants. *p*-value < 0.0001. The results are presented in Table 3.

Lumbar spine flexion control test in the sitting position—knee extension, *n* = 23. The test results showed failure to meet quantitative and qualitative criteria (X, X) in 15 participants, failure to meet the qualitative aspect while meeting the quantitative aspect (V, X) in 2 participants, and satisfaction of both assessment aspects (V, V) in 6 participants (*p*-value < 0.0001). The results are presented in Table 3.

Lumbar spine rotation control test in the sitting position—knee extension, right leg, *n* = 23. The test results showed failure to meet quantitative and qualitative criteria (X, X) in 10 participants, failure to meet the qualitative aspect while meeting the quantitative aspect (V, X) in 3 participants, and satisfaction of both assessment aspects (V, V) in 10 participants (*p*-value < 0.0001). The results are presented in Table 3.

Control test of rotation of the lumbar spine in the sitting position—knee extension, left leg, *n* = 23. The test results showed failure to meet quantitative and qualitative criteria (X, X) in 9 participants, failure to meet the qualitative aspect while meeting the quantitative aspect (V, X) in 5 participants, and satisfaction of both assessment aspects (V, V) in 9 participants (*p*-value < 0.0001). The results are presented in Table 3.

Lumbar spine flexion control test in the sitting position—forward leaning, *n* = 23. The test results showed failure to meet quantitative and qualitative criteria (X,X) in 13 participants, failure to meet the qualitative aspect while meeting the quantitative aspect (V, X) in 2 participants, and satisfaction of both assessment aspects (V, V) in 8 participants (*p*-value < 0.0001). The results are presented in Table 3.

**Figure 9:**
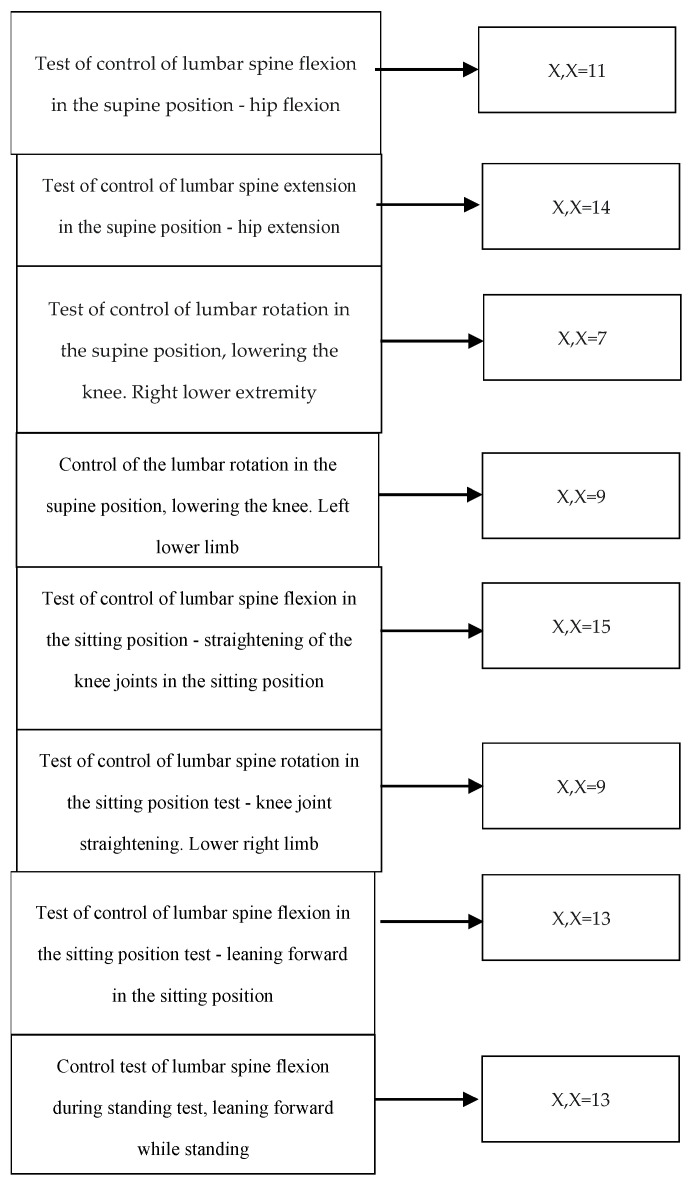
Summary of directional lumbar motor control testing results. Score X, X—impaired directional control of lumbar movement. *n* = 23, *p*-value < 0.0001.

Flexion control test of the lumbar spine in standing position—forward leaning while standing, *n* = 23. The test results showed failure to meet quantitative and qualitative criteria (X, X) in 13 participants, failure to meet the qualitative aspect while meeting the quantitative aspect (V, X) in 3 participants, and satisfaction of both assessment aspects (V, V) in 7 participants (*p*-value < 0.0001). The results are presented in Table 3. Figure 9 presents a summary of lumbar spine directional control errors in the study group. Table 4 presents a summary of the percentage results of the directional assessment of the lumbar spine in the study group.

## 4. Discussion

Special forces carry out combat operations using specialized and unconventional forms of combat compared to regular army formations. These operations are highly risky for injury and trauma. The most frequently reported complaint is low back pain. Controlling lower body movement depends on the stability of the lumbar–pelvic–hip complex and is critical to preventing low back pain syndromes. As special forces soldiers are exposed to high levels of overuse and musculoskeletal injuries, they are a priority group for injury prevention. Motor control assessment activities can potentially be necessary to minimize the incidence of overuse and injury among special forces soldiers if prevention strategies are to be developed. Introducing physiotherapy care will allow continuous monitoring and early intervention to prevent injury [4,6].

There is a lack of direct reports in the available literature that evaluate the lumbar spine area using movement dissociation testing in a group of special forces operators. The deficiencies in the available literature provided motivation to work to fill the existing literature gap. The work is a pilot study that provides an introduction to the validity of the application of the assessment of motor control of the lumbar spine in the described group, where the available literature is scarce. Exploring issues related to the evaluation and reeducation of motor control disorders in the defined group also aims to improve the quality of physiotherapy care so that it becomes more effective and, in the long term, may potentially contribute to improving the quality of life of operators.

A detailed history and examination are central to functional diagnosis. In the study group, 69.57% reported lumbar pain three months before evaluation. At the same time, the lumbar spine was the most common site of pain in the study group compared to other parts of the body. These findings are consistent with reports from different authors. In their systematic review, Stannard and Fortington report that the lumbar spine, knee joints, and ankle joints are the most common sites of pain [6]. Lendal and Kjaer, on the other hand, suggest the shoulder girdle, lumbar spine, and knee joints as the most common localized areas of pain [22].

Given the above information, it seems reasonable to pay special attention to the motor assessment elements as part of the physical examination, therapy, and prevention of lumbar spine pain syndromes in comprehensive medical care in a group of Special Forces operators. Of course, it is essential to recognize that some complaints of lumbar pain may be related to direct injuries sustained during specialized training, such as parachute jumping, and this type of injury cannot obviously be prevented by a physiotherapist [6]. On the other hand, some injuries are caused by a mechanism of accumulation of microtraumas that eventually leads to structural damage and sudden onset of symptoms, such as acute discopathy (21). We should also not forget the possibility of the formation of spinal overload syndromes not associated with direct trauma but resulting from the long-term accumulation of overload related to the intensive nature of the service. It is associated with the possibility of a slow build-up of symptoms over a long period of time without an acute onset of pain [23,24]. Stannard and Fortington identify physical training as the most common cause of injury and pain in the described group [6]. Therefore, it seems reasonable to introduce some elements of motor training as part of developing the operators’ fitness and elements of targeted motor assessment in the framework of physiotherapy care as part of the optimization of movement tailored to the individual soldier. It is necessary to minimize the risk of lumbar pain syndromes associated with the accumulation of overload and the resulting incidents of lumbar pain, which can cause operators to be removed from duty. Therefore, an essential element in the process of motor reeducation is the personalization of the improvement program [13] in terms of the reeducation of existing motor deficits. It makes it possible to use elements of motor control testing in physiotherapeutic diagnosis, including movement dissociation tests based on the Kinetic Control physiotherapeutic method [13,25]. An accurate motor assessment would allow one to create a detailed motor profile of each operator so that the subsequent motor training or rehabilitation process would respond to specific motor errors. On the other hand, the development of research in this area in a larger group of subjects would potentially allow the creation of general guidelines according to the specifics of the motor training elements performed in motor training in the field of musculoskeletal prevention for the target group, that is, operators of special units.

### 4.1. Summary of Results

The aim of the study was to evaluate directional control of the lumbar spine using the dissociation tests included in the Kinetic Control physiotherapy method. The results obtained in this study indicate a statistically significant appearance of motor control disorders in the form of impaired control of movement (X, X result) in the evaluated tests (*p*-value < 0.0001). Among the study group of *n* = 23, failure to meet the quantitative and qualitative criteria assessed that confirmed the presence of motor control impairment as uncontrolled movement was observed in the following cases: 11 participants in the supine lumbar flexion control test, 15 participants in the lumbar flexion control test in the sitting position during knee extension, 13 participants in the lumbar flexion control test in the sitting position during forward leaning, 13 participants in the lumbar flexion control test in the standing position during forward leaning, 7 and 9 participants in the lumbar rotation control tests in the supine position while lowering the right and left lower limbs, respectively, 9 and 10 participants in the lumbar rotation control tests in the sitting position during knee extension of the left and right lower limbs, and 14 participants in the lumbar extension control test in the supine position during hip extension.

### 4.2. Interpretation of Results and Clinical Implications

These reports confirm impaired control of movement of the lumbar spine during hip and knee joint movement in the sagittal and transverse planes. The results of the motor control examination identify the existence of directional motor control deficits in the lumbar spine area in a group of army special units operators. These reports are an attempt to fill the existing gaps in the field of scientific reports on the assessment of motor control of the lumbar spine area. In terms of clinical practice, the information obtained directs to the necessity of this type of evaluation especially in groups of people subjected to heavy loads in the motor area, where such a method of evaluation is not a standard element as well as confirms the occurrence of such disorders. The results obtained can also provide a basis for implementing elements of reeducation of directional motor control in the lumbar spine area based on low-threshold training that rebuilds the control mechanisms of this area.

In the case of uncontrolled lumbar flexion and rotation, there is a potentially higher risk of overloading structures in the posterior column of the spine concerning the components of the motor segment, i.e., the ligamentous and intervertebral disc system or the posterior part of the intervertebral disc [26,27]. This condition is associated with an abnormal functioning of the multifidus muscle in the lumbar spine [28,29]. Uncontrolled rotation will further promote asymmetric loading of the posterior column of the spine, as previously described, for uncontrolled flexion of the lumbar spine [26,27]. The flexion-rotation mechanism in the lumbar spine is one of the most common mechanically contributing mechanisms to discopathy [30]. On the other hand, uncontrolled upright movement of the lumbar spine may be associated with excessive stress on the interarticular joint area, resulting in pain [31]. This condition is related to the abnormal interaction of the anterior abdominal wall muscles with the gluteus maximus muscles to control the lumbopelvic area [32].

It is also worth mentioning that before all the tests were performed, the available passive range of motion of the hip joints and the flexibility of the ischiofemoral muscles were assessed to check if the subject could be subjected to a motor assessment of range of motion, referred to by the test authors as the benchmark [13]. The appearance of errors in the form of movement dissociation disorders is described as a consequence of X, X and is associated, among other things, with the dysfunction of the muscles responsible for movement control and not with the presence of limitations in passive mobility of the hip joint or limited flexibility of the ischiofemoral group. Therefore, motor errors that occur are not the result of existing tissue limitations but of a disturbance in motor control elements, which should undergo a reeducation process, especially in a group exposed to high levels of musculoskeletal stress. A group of special unit operators should undergo a detailed physical therapy assessment, particularly motor assessment, to identify motor deficits. The appearance of motor control deficits, combined with repeated movements that involve trunk flexion performed in full body armor during many aspects of training, could be a significant factor causing lumbar spine pain in the described group. The results confirm the presence of motor control deficits in the lumbar spine area in the evaluated group. In the scope of physiotherapeutic care intended for operators of special units, this element of evaluation should be introduced. Based on the results of motor control evaluation, the improvement process based on optimization of movement patterns and reeducation of uncontrolled movement can be programmed. Elements of evaluation directional motor control of lumbar spine should be the starting point for planning the motor reeducation process and should be integrated into the physiotherapeutic care of a group of operators exposed to high levels of stress in the lumbar spine area. It seems worthwhile to introduce optimization of movement patterns in terms of reeducation of lumbar directional control as a permanent element of physiotherapy care in the evaluated group. Based on the results of the motor control assessment obtained in the study, as part of movement optimization, in addition to incorporating this type of assessment into the way this group is evaluated, it would also be worthwhile to implement targeted therapeutic elements. These elements should be directed at activating the multifidus muscle, the abdominal muscles and the gluteal muscles in the lumbar region for improved control of the direction of flexion and rotation movement, as well as the abdominal muscles with the gluteal muscles for control of the direction of upright movement. The starting point for the beginning of the process of reeducation of motor control can be to conduct training in the range of movements used in the described tests until a result indicating that the quantitative and qualitative parameter of testing is met. However, with regard to the described group of special unit operators, it should be remembered that such training should later be gradually developed with an appropriate level of progression due to the previously indicated high level of loads to which the soldiers’ musculoskeletal system is subjected.

### 4.3. Limitations and Future Development of Research

This study is a pilot study, and the results obtained, which are preliminary reports, indicate the occurrence of lumbar motor errors in the described special forces operator group. It is necessary to carry out a study on a larger group of operators. The small number of subjects constitutes a limitation of the research work. However, the strength of the work is its innovation, which points to the fact that there are control deficits in the form impaired control of movements in the area of the lumbar spine in this group. It is necessary to continue and extend research in this direction Another limitation of the above study is that the evaluation was conducted on a specific group of subjects. From one perspective, the group of operators of special units is a group that should be subjected to physiotherapeutic care covering the widest possible spectrum of influence due to the subjection of soldiers to a high level of stress and a specific and multidirectional training process. However, precisely because of the high level of specificity of the preparation process and the duties of the operators performed, the results obtained cannot be applied to the general population.

This study based on evaluation using movement dissociation tests is a starting point for further research work in the area of motor control deficits and needs to be developed also in terms of the evaluation tools used. The study was able to identify motor control deficits of the lumbar spine. The authors’ choice of motor control tests was also dictated by their desire to use evaluation components that are commonly available in physiotherapeutic functional assessment settings. This work represents a pilot study in terms of research development, it would be worthwhile to supplement motor control assessment with kinematic analysis of the lumbar spine using digital motion analysis systems in the future.

Given the above, it seems essential to extend comprehensive physiotherapeutic care to operators of special units, in which great emphasis should be placed on the prevention of the formation of overload syndromes of the lumbar spine, with the possible inclusion of evaluation of motor control as one of the potential elements of the therapeutic process. One of the tools used to address this issue is motor control training. Training should be preceded by a detailed assessment of motor control as a starting point for planning and implementing a personalized improvement program.

Implementing reeducation in motor control disorders, general motor preparation training, adequate physical training preparation, education in kinesiology and ergonomics, or care for multidirectional wellness of the operator are all necessary elements to keep the operator fully healthy and fit despite the high levels of stress on the body.

## 5. Conclusions

The main results of the present study showed the presence of uncontrolled functional patterns of the lumbar spine in a group of special unit operators. Impaired control of movement was observed in the direction of lumbar flexion, rotation, and extension, which may be potentially associated with the generation of lumbar spine pain syndromes. Identifying the occurrence of uncontrolled movement allows physiotherapists to monitor the state of lumbar motor control. Physiotherapists can use the results obtained from the assessment to improve therapy programming and prevention and improvement of therapeutic management.

## Figures and Tables

**Table 1:** Basic demographic data and declared service duration in combat units of the study group (*n* = 23).

Age [Years]	Height [cm]	Weight [kg]	Body Mass Index (BMI)	Declared Service Time in Combat Units [Years]
40.26 ± 4.5	178.9 ± 6.25	84.57 ± 8.0	26.40 ± 1.45	8.54 ± 6.0

**Table 2:** Pain locations in the musculoskeletal system (% of participants reporting pain in a given area in the last 3 months).

Reported Pain Location	Respondents Reporting Pain [%]
Lumbar spine	69.57
Knee area	60.87
Shoulder girdle	52.17
Foot and ankle	30.43
Hip joint area	26.09
Head and cervical spine	21.74
Thoracic spine and ribs	21.64
Elbow Complex	13.04
Wrist and hand	8.70

**Table 3:** Results of motor control tests of the lumbar spine (*n* = 23).

Test of control of lumbar spine flexion in the supine position—hip flexion				
*n* = 23	X, X	V, X	V, V	*p*-value < 0.0001
Result	11	8	4	
Test of control of lumbar spine extension in the supine position—hip extension				
*n* = 23	X, X	V, X	V, V	*p*-value < 0.0001
Result	14	4	5	
Test of control of lumbar rotation in the supine position, lowering the knee. Right lower extremity				
*n* = 23	X, X	V, X	V, V	*p*-value < 0.0001
Result	7	4	12	
Control of the lumbar rotation in the supine position, lowering the knee. Left lower limb				
*n* = 23	X, X	V, X	V, V	*p*-value < 0.0001
Result	9	4	10	
Test of control of lumbar spine flexion in the sitting position—straightening of the knee joints in the sitting position				
*n* = 23	X, X	V, X	V, V	*p*-value < 0.0001
Result	15	2	6	
Test of control of lumbar spine rotation in the sitting position test—knee joint straightening. Lower right limb				
*n* = 23	X, X	V, X	V, V	*p*-value < 0.0001
Result	9	5	9	
Test of control of lumbar spine flexion in the sitting position test—leaning forward in the sitting position				
*n* = 23	X, X	V, X	V, V	*p*-value < 0.0001
Result	13	2	8	
Control test of lumbar spine flexion during the standing test, leaning forward while standing				
*n* = 23	X, X	V, X	V, V	*p*-value < 0.0001
Result	13	3	7	

**Table 4:** The results of motor control tests of the lumbar spine (*n* = 23).

Lumbar flexion control test in the supine position—hip flexion			
	XX	VX	VV
Result [%]	47.83	34.78	17.39
Lumbar flexion control test in the supine position—hip extension			
	XX	VX	VV
Result [%]	60.87	17.39	21.74
Lumbar rotation control test in the supine position—lowering the knee. Right lower limb			
	XX	VX	VV
Result [%]	30.43	17.39	52.17
Lumbar rotation control test in the supine position—lowering the knee. Left lower limb			
	XX	VX	VV
Result [%]	39.13	17.39	43.48
Lumbar flexion control test in the sitting position—straightening the knee joints in the sitting position			
	XX	VX	VV
Result [%]	65.22	8.70	26.09
Rotation control of the lumbar spine in the sitting position test—straightening of the knee joint. Right lower limb			
	XX	VX	VV
Result [%]	43.48	13.04	43.48
Rotation control of the lumbar spine in the sitting position test—straightening of the knee joint. Left lower limb			
	XX	VX	VV
Result [%]	39.13	21.74	39.13
Control of lumbar flexion in the sitting position test—leaning forward in the sitting position			
	XX	VX	VV
Result [%]	56.52	8.70	34.78
Control of lumbar flexion in the standing test—leaning forward while standing			
	XX	VX	VV
Result [%]	56.52	13.04	30.43

## Data Availability

The data can be provided upon a reasonable request to the corresponding author.
